# Supplementary material for: Identification of Freezing-Responsive microRNAs and Their Targets in Chinese Jujube by Small RNA and Degradome Sequencing
Source: Int J Mol Sci. 2026 Sep 4;27(17):7912. doi: 10.3390/ijms27177912 (PMC13566890; doi:10.3390/ijms27177912)
Supplement: Supplementary file 1 [file ijms-27-07912-s001.zip › Supplementary Material S2/novel_miR_177.pdf]

Provisional ID	:	novel_miR_177
Score total	:	1.5
Score for star read(s)	:	-1.3
Score for read counts	:	0
Score for mfe	:	1.2
Score for randfold	:	1.6
Score for cons. seed	:	
Total read count	:	95
Mature read count	:	55
Loop read count	:	40
Star read count	:	0

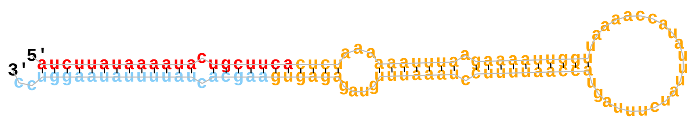

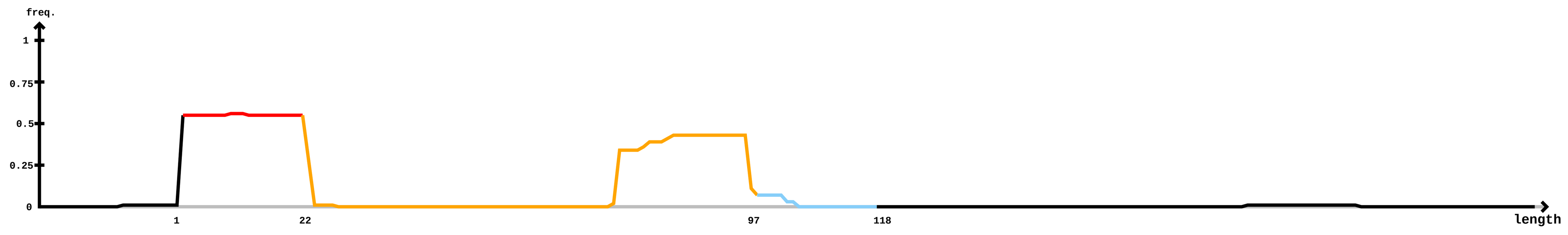

Mature

Star

5'- cugcuauacgagcaagaaaacgaucuuauaaaauacugcuucacucuaaaaaaauuuaagaaaauugguuaaaaccauauuuaucuuuaguaccaauuuuccuaaauuuguagagagugaagcacuauuuuauaagguccuuuucuugcuacuuauuauuauuauuauuauuauuguuuuaugaaaccuuuuuuucuuguuaauuggaaauaugggguuguuacaaaaauugauuaauuaauaauuaauuaaaug -3' exp

.........((((((((.(((((((((((((.((((((((((...((((((.(((((((((....................)))))))))).)))))))....)))))))))).))))))))))))).))))))))))))))))).))))))))))..................((((......((((...(((((.......)))))...)))))...))))...(((((((((...)))))))))...

read	reads	mm	sample
caagaaaacgaucuuauaaaa	1	0	LT-3
aucuuauaaaauacugcuuca	30	0	LCK-1
aucuuauaaaauacugcuuca	24	0	LCK-2
aaauacugcuucacucua	1	0	LCK-3
auuuuccuaaauuuguagagagug	2	0	LCK-3
uuuuccuaaauuuguagagCgu	1	1	LCK-2
uuuuccuaaauuuguagagagu	11	0	LCK-1
uuuuccuaaauuuguagagagu	20	0	LCK-2
ccuaaauuuguagagagug	2	0	LT-1
cuaaauuuguagagagugaagca	1	0	LT-1
cuaaauuuguagagagugaagcacu	1	0	LT-3
cuaaauuuguagagagugaagcacu	1	0	LT-1
aauuuguagagagugaagca	1	0	LT-1
aauuuguagagagugaagcacu	1	0	LT-3
auuuguagagagugaagca	2	0	LT-2
uggaaauaugggguuguuac	1	0	LT-3
